# Treatment Adherence and Quality of Life of Adults Living With Hypertension in Rural Ghana

**DOI:** 10.1002/nop2.70198

**Published:** 2025-03-23

**Authors:** Ayisha Blessing Amadu, Kennedy Dodam Konlan, Jamilatu Barbara Amadu, Gladys Dzansi

**Affiliations:** ^1^ Department of Nursing College of Nursing and Midwifery Nalerigu Ghana; ^2^ Department of Adult Health, School of Nursing and Midwifery University of Ghana Legon Ghana; ^3^ St. Michael's Nursing and Midwifery Training College Pramso Ghana

**Keywords:** hypertension, quality of life, rural Ghana, treatment adherence

## Abstract

**Aim:**

We determined treatment adherence and quality of life of adults living with hypertension in rural Ghana.

**Design:**

A quantitative cross‐sectional survey was conducted.

**Method:**

We randomly sampled 351 persons diagnosed with hypertension at the Baptist Medical Centre in Nalerigu, Ghana. A shortened version of the World Health Organization's Quality of Life (WHOQOL‐BREF) questionnaire was used, and information on treatment adherence was collected using a modified Morisky Medication Adherence Scale. Data analyses were conducted with the aid of SPSS 23.0 at a 95% confidence level.

**Results:**

The study revealed a high rate of treatment adherence, 226 (64%) with low quality of life among the participants. Adherence to treatment was more associated with the males compared to females. The social health dimension of the quality of life was significantly associated with treatment adherence. The role of social networks in enhancing adherence was a major finding in this study and could be harnessed by nurses and midwives to improve the lives of persons diagnosed with hypertension. It is recommended that measures that promote remembrance and reduce forgetfulness regarding hypertension medications, such as mobile health and digital technologies, be implemented by health workers to enhance treatment adherence. Further, measures aimed at improving resource allocation for women in rural communities, such as women‐economic empowerment programmes, are encouraged to enhance health‐seeking and treatment adherence.

**Patient or Public Contribution:**

None.

**Abbreviations**
BMC: Baptist Medical Centre
BP: blood pressure
CHAG: Christian Health Association of Ghana
CVDs: cardiovascular diseases
GHS: Ghana Health Service
HPT: hypertension
NCDs: non‐communicable diseases
OPD: out‐patient department
PLWHPT: people living with hypertension
SSA: sub‐Saharan Africa

## 1 Introduction

Hypertension, also known as high blood pressure (BP), has been acknowledged widely as the leading cause of global‐illness‐burden (Atibila et al. 2021; Konlan, Afam‐adjei, et al. 2020; World Health Organization [WHO] 2020), with an increasing prevalence, particularly in low‐ and middle‐income countries (Atibila et al. 2021; Konlan, Baku, et al. 2020). Almost 2 billion people globally live with hypertension and this is expected to rise exponentially (Atibila et al. 2021; Konlan, Afam‐adjei, et al. 2020; WHO 2020). Hypertension is described as a systolic blood pressure of ≥ 130 mmHg and diastolic blood pressure of ≥ 80 mmHg by the American College of Cardiology (ACC) and the American Heart Association (AHA) in 2017 (Konlan, Afam‐adjei, et al. 2020). Hypertension is a prime medical condition that dramatically increases the risk of brain, kidney, heart and other disorders (Atibila et al. 2021; Aziato et al. 2021; Konlan et al. 2023) in victims. Hypertension is primarily asymptomatic and this can cause individuals to undermine the need for treatment, especially in younger, more active individuals without any comorbidities, who may have never experienced adverse effects, or find it difficult to fit any treatment into their schedule (Konlan et al. 2023). Since hypertension develops into its complicated form without exhibiting any signs, it is sometimes known as ‘a silent killer’ (Konlan, Afam‐adjei, et al. 2020; WHO 2020). The disease's nature makes it challenging to get an early diagnosis before it causes numerous cardiovascular and other consequences if screening is not done regularly (Konlan et al. 2023). In line with this, complications are often present when hypertension is diagnosed, especially in developing nations where people are less likely to seek out a general routine physical examination at least twice annually (Konlan, Baku, et al. 2020).

Reducing the negative consequences of high blood pressure (BP) requires that persons diagnosed with high blood pressure adhere to their prescribed treatment and management instructions (Atibila et al. 2018; Konlan et al. 2023; Nyaaba et al. 2018). The current biomedical model of care requires that patients with hypertension, aside from lifestyle modifications, take in prescribed drugs to reduce their rising BPs (Atibila et al. 2021; Konlan et al. 2023; Nyaaba et al. 2018). Adherence to treatment is crucial in preventing hypertension‐related complications (Atibila et al. 2021; Konlan, Baku, et al. 2020) and has been shown to improve quality of life and reduce primary care, outpatient and emergency department visits (Konlan et al. 2023; Nyaaba et al. 2018).

The Ministry of Health, Ghana, through the Ghana Health Service (GHS) ensures that individuals diagnosed with high BPs receive quality allopathic care that will contribute to reducing the occurrence of complications (Konlan et al. 2023). Thus, specialised care is proximally available for managing hypertension in most urban and peri‐urban parts of Ghana (Atibila et al. 2021). However, persons diagnosed with high BPs from the rural communities in Ghana travel several kilometres to access health care, and this could affect their level of adherence (Konlan et al. 2023). In addition, most individuals with high BPs in Ghana might not be aware of the great benefit of treatment adherence, particularly on their quality of life, having been diagnosed with the chronic situation of high BPs, and may contemplate suicide (Aziato et al. 2021; Nyaaba et al. 2018; Konlan et al. 2020c). The suicidal tendencies in hypertensive persons in Ghana, particularly, have been reported by some authors (Aziato et al. 2021; Nyaaba et al. 2018) as largely due to the emotional burden the disease places on the victims due to the chronicity of the condition and the financial implications of the same as well as the deteriorating effects of the disease, and its drugs on the activities of daily living of patients, particularly their sexual function.

Health‐related Quality of Life (HRQoL) is increasingly recognised as being essential to patient overall outcomes (Aziato et al. 2021; Sitlinger and Zafar 2018). An individual's quality of life is negatively impacted by hypertension (Ugwuja et al. 2015; Wang et al. 2018; Zhang et al. 2017), and the significant incidence of hypertension and general lack of awareness worsens the situation (Zhang et al. 2021). Previous research (Konlan et al. 2023; Wang et al. 2018; Zhang et al. 2021) has also demonstrated that the presence of patient health comorbidities, awareness of the diagnosis and negative side effects of medications are all associated with a considerable decline in the HRQoL of people living with hypertension (PLWHPT). In addition, a third of patients with comorbidities and nearly half of hypertension patients do not take their medications as prescribed (Ugwuja et al. 2015; Zhang et al. 2021) even though available evidence demonstrates that antihypertensive medication is effective in reducing blood pressures in individuals for approximately 4 years on average (Canoy et al. 2022). Further, the literature suggests a decrease in fatalities and cardiovascular complications with treatment adherence in other jurisdictions (Zhang et al. 2021) with a reported improvement in health outcomes as well as overall quality of life (Wang et al. 2018).

Hypertension is linked to a slew of undesirable traits and subsequent complications that have a detrimental impact on the quality of life (Kretchy et al. 2019). Several studies have shown that being aware of a chronic disease can damage the health‐related quality of life (HRQoL) and may have psychological effects on the individual greater than the disease itself (Aziato et al. 2021; Sang et al. 2021). Hypertension, as a leading cause of cardiovascular illness, has been linked to a reduction in the Quality of Life (QoL), particularly concerning the elderly (Canoy et al. 2022; Konlan et al. 2023; Kretchy et al. 2019) and that enhancing treatment adherence is key to improving the health‐related quality of life in persons living with high BPs (Konlan et al. 2023; Wang et al. 2018; Zhang et al. 2021). However, it remains largely undetermined the relationship between treatment adherence and the quality of life of persons being managed for hypertension in the rural part of Ghana.

### 1.1 Aim

We determined treatment adherence and quality of life of adults living with hypertension in rural Ghana.

### 1.2 Conceptual Framework Underpinning the Study

**FIGURE 1:**
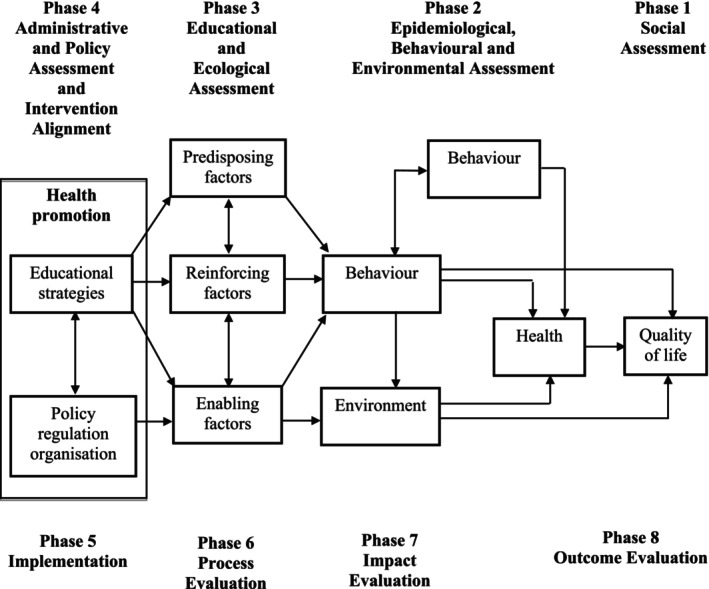
Healthcare utilisation model.

A person's level of quality of life and adherence to treatment can be evaluated using a variety of models. Three models, in addition to the PRECEDE model utilised in this study, were initially considered: the Health Belief Model (HBM), the chronic care model (1990) and the Ferrans and colleagues Health‐Related Quality of Life model; their constructs, however, proved to not adequately define the objectives of this study and, as such, they were deemed less than ideal for this study. Taking the aim and objectives of this study into consideration, the PRECEDE model was the most relevant to the study as it was applicable and practical to all aspects. A schematic illustration of the PRECEDE model is shown in Figure 1.

## 2 Method

### 2.1 Study Design

The study adopted a quantitative approach using a cross‐sectional survey design. The cross‐sectional survey design was suited for this study because data were obtained during a particular period with no follow‐up on participants.

### 2.2 Setting

The study was conducted at the Baptist Medical Centre (BMC), a well‐known mission hospital in Nalerigu, the Northeast regional capital of Ghana, and serves as a referral point for rural persons living with hypertension in the north‐eastern part of Ghana. It is the largest hospital located in the North‐Eastern part of rural Ghana, with the greatest number of out‐patient department (OPD) attendances for people living with hypertension (PLWHPT) in the region. It is also the most equipped hypertension management facility in the region. Every workday, between 200 and 400 patients are seen, and information from the Biostatistics Department of the Hospital (BMC) has shown hypertension to be consistently among the top 10 causes of hospital attendance among adults.

### 2.3 Sample Size Determination

The sample size was estimated at a 95% confidence interval to be 325 using the Cochran method, which has successfully been used in numerous studies worldwide and is arguably the most popular sample size calculation method. A 10% of increase in the sample size was then made to account for non‐responses, which brought the number to 357. Patients were included in the study if they met the following inclusion criteria: only patients diagnosed with hypertension for over 6 months from rural communities, participants who were rural residents (staying outside the regional capital of Nalerigu), participants without any other disease condition, and were conscious to be able to give voluntary consent. Patients who were critically ill/diagnosed with other serious diseases like stroke, less than 6 months’ diagnosis of hypertension, residents of Nalerigu township, and those with other comorbidities or too old or weak to take part in the study were excluded from the study.

A simple random sampling procedure was conducted using the patient folders of those with hypertension by balloting them without replacement at the OPD of the BMC. To begin with, a numbered list of all patients presenting with hypertension was created and used as a sampling frame. With the aid of a three‐digit random number table, the first 357 participants were selected. If during the researchers administering the questionnaire, a participant dropped out, refused to consent or was unreachable, the next available random number and its equivalent hypertensive patient were contacted. This process continued until a total of 357 participants were selected. Data were collected between June 3 and July 31, 2022.

### 2.4 Selection of Respondents and Data Collection

Data collection took place in the months of June and July 2022. This was after ethical approval was granted by the Christian Health Association of Ghana (CHAG), and permission was given to collect data by the management of BMC. Even though the original tools for the study were in the English language, this was translated into Mampruli and Hausa, which are local/native languages largely spoken in that part of Ghana. The translation of the tool was with the aid of a language specialist fluent in both English and the languages of interest (Mampruli and Hausa). A graduate nurse, fluent in English, Mampruli and Hausa, was enlisted and trained to assist with the data collection process. Considering that most of the participants were not literate, the research assistant assisted the researchers who were also fluent in English, Hausa and Mampruli to an administer the questionnaire. The researchers took time to explain the study's goal and the data collection tool and appropriately addressed all concerns. The assistant helped in physically identifying clients who matched the study's inclusion criteria. Eligible candidates were then approached by the investigators for written informed consent. On each day, all individuals who matched the inclusion requirements were addressed. During the recruitment session, the goal of the study was presented in detail to potential participants to get their cooperation. Each participant's right to withdraw from the study without any repercussions was also emphasised. Participants who afterward showed interest in partaking in the study were given the information sheet to read. In instances where participants could not read, the information sheet was read to them or explained in Hausa or Mampruli or their preferred language that they understood and then guided to sign the written informed consent form. Those who were unable to sign were provided with an ink pad to thumbprint their approval.

The data collection was done using a pre‐tested questionnaire. The questionnaire contained information on the socio‐demographic variables such as age, sex, level of education, duration of being diagnosed with hypertension, among others. The questionnaire also collected information on treatment adherence using a modified Morisky Medication Adherence Scale (MMAS) which had been used in a similar population in the urban part of Ghana (Konlan et al. 2023) and health‐related quality of life using the World Health Organisation's health‐related quality of life brief questionnaire (WHOQOL‐BREF).

The Morisky Medication Adherence Scale is among the most commonly used patient questionnaires for assessing medication adherence (MMAS) (Konlan et al. 2023). Originally a 4‐item instrument but now 8‐items, it has historically been used in randomised studies of medication adherence strategies among patients with a range of chronic diseases. The 8‐item Morisky Medication Adherence Scale (the amended version) was intended to address some of the shortcomings of the original scale (MMAS‐4) by adding four more items that addressed the conditions underlying adherence behaviour. Seven of the eight items on the MMAS‐8 are yes‐or‐no questions, and one of them uses a 5‐point scale (rating on a 5‐point scale) (Morisky et al. 2008). The scale is reliable with a Cronbach *α* of 0.83; the MMAS‐8 has been demonstrated to be trustworthy in assessing adherence in hypertensive individuals (Konlan et al. 2023; Morisky et al. 2008). Thus, the current study used MMAS‐8 with a Cronbach's *α* of 0.83 to collect information on medication adherence.

Regarding the quality of life, a shortened version of the WHOQOL‐100 questionnaire called the WHOQOL‐BREF was used to assess the quality of life of the participants. It is a self‐administered research instrument that includes 26‐items about an individual's health and well‐being in the past 2 weeks. Questions are answered on a 1–5 Likert scale, with 1 indicating ‘disagree’ or ‘not at all’ and 5 indicating ‘totally agree’ or ‘extremely’. Also, it is divided into four domains, each with its own set of features (Kalfoss et al. 2021). The domains include physical health, psychological, social relationships and the environment. Additionally, there are two distinctive questions, one about the individual's overall impression of their health and the other about the individual's general impression of the actual quality of life. The WHOQOL Group in 1998 stated that in the event that time is a constraint, the WHOQOL‐BREF resonates well with the WHOQOL‐100 indicating that it is a viable option to the lengthier version. Additionally, the WHOQOL‐BREF has been determined to be a robust trustworthy assessment of QoL across various cultures (Kalfoss et al. 2021). Cronbach *α* values for every one of the four domain scores for the WHOQOL questionnaire used in this study ranged from 0.66 to 0.84, indicating excellent internal consistency.

Each item on the questionnaire was read and interpreted to the participants by the researchers, and the participants' responses were ticked (researcher administered). For participants who were not comfortable with the English language, each item on the questionnaire was read in English (as written on the questionnaire), translated to Hausa or Mampruli (or any other Ghanaian language they were more comfortable with) and re‐translated back to English to ascertain responses. A field notebook was used in detailing how each piece of information was logged. Data management began at the data‐collecting site, with a check for missing information. The participants were recruited daily until the total number of participants required for the study was reached.

### 2.5 Data Analysis

Data were loaded into the Statistical Package for the Social Sciences (SPSS) version 23.0. Data cleaning was done by means of visual inspection and running descriptive analysis, particularly frequencies and percentages, as well as means and standard deviations where appropriate. Specifically, variables such as sex, marital status, educational level, religion and occupation, which were categorical, were presented as frequencies and percentages. The scores on the adapted scale were summed to create an overall adherence score with a possible score range of 0–8. Scores of 6 and above were considered high adherence, representing adherence to treatment, whereas scores below 6 were considered low levels of adherence, representing non‐adherence. Regarding the quality of life, we analysed the WHOQOL tool, which was measuring the quality of life using a 5‐point Likert scale, with figures in the continuum 1–5 showing increasing agreement with participants' QoL metrics. The 5‐point Likert codes were used as scores, and composite scores were generated for every participant by adding the scores for every QoL component question for each domain of quality of life (physical, psychological, social and environmental). We then found the mean and SD of the responses for each domain of the quality‐of‐life scale. The relationship between treatment adherence and the socio‐demographic variables was determined using chi‐squared analysis, as most of the socio‐demographic variables were categorical. We further determined the association between quality‐of‐life domains and treatment adherence with a level of significance of < 0.05.

## 3 Results

### 3.1 Socio‐Demographic Characteristics of Participants

Three hundred and fifty‐one participants with hypertension took part in this study. The average age of participants was approximately 60 years (*M* = 59.8, SD = 14.18). More than half (54.90%) of the participants were females. Each participant had an average of almost seven children (*M* = 6.62, SD = 3.49) with the minimum being one (1) child and a maximum of 20 children. Also, almost 40% (39.03%) of the participants were married with 39.89% being believers of the Islamic religion. Almost 60% (59.54%) claimed that they had formal education with 65.24% claiming to be earning above the minimum wage in Ghana. Further, 66.38% were fully employed as depicted in Table 1.

**TABLE 1:** Socio‐demographic characteristics of participants.

Socio‐demographic characteristics	Frequency (*n*)	Percentage
	351	100
**Sex**		
Male	160	45.60
Female	191	54.40
**Marital status**		
Single	98	27.92
Married	137	39.03
Divorced/Separated/Widowed	116	33.05
**Religion**		
Christianity	112	31.91
Islam	140	39.89
African traditional religion	99	28.77
**Educational status**		
No formal education	142	40.46
Formal education	209	59.54
**Monthly income level**		
Less than GHC 350 (less than minimum wage)	122	34.76
More than GHC 351 (more than the minimum wage)	229	65.24
**Employment status**		
Fully employed	233	66.38
Unemployed/retired	118	33.61

### 3.2 Adherence to Treatment Among Participants

The study results found that 226 (64.4%) of participants had high adherence to medication, and 125 (35.6%) of participants had low adherence to hypertensive medication as shown in Table 2.

**TABLE 2:** Level of medication adherence.

Level of medication adherence	Frequency (*n*)	Percentage
Low adherence	125	35.6
High adherence	226	64.4
Total	351	100

### 3.3 Reasons for Medication Non‐Adherence Among the Participants

The participants were asked to enumerate the factors that caused the participants who did not adhere to medication not to adhere to the hypertension medication; the factors they listed included occasional forgetfulness, inconvenience, lack of trust in medications, too many side effects and better outcomes from herbals. Table 3 gives the details about the responses for the non‐adherence.

**TABLE 3:** Reasons for medication non‐adherence among the participants.

Reasons for medication non‐adherence	Frequency (*n*)	Percentage
	125	100
Occasional forgetfulness	96	76.8
Inconvenience	78	62.4
Lack of trust in the medication	102	81.6
Too much side effects of drugs	86	68.8
Better outcome from herbals	93	74.4

### 3.4 Quality of Life of Participants

The results in Table 4 show that even though the overall quality of life was found to have a very high mean score (91.46 ± 8.54), the various domains of the quality of life had very low mean scores indicative of poor quality of life. The social domain of the quality of life had the lowest mean score of 10.69 ± 2.07 compared to the environmental domain with the highest mean quality of life score of 30.34 ± 3.88 (Table 4).

**TABLE 4:** Descriptive statistics of WHOQoL scores measuring the quality of life of participants.

Participants' quality of life (QoL)	Mean ± standard deviation	Skewness	SE of kurtosis (SEK)
Overall quality of life as reported by participants	91.46 ± 8.54	0.056	0.130
WHOQoL domains			0.130
Physical	25.22 ± 3.19	−0.16	0.130
Psychological	21.62 ± 4.08	−0.35	0.130
Social	10.69 ± 2.07	0.197	0.130
Environmental	30.34 ± 3.88	0.126	0.130

### 3.5 Association Between Medication Adherence and Socio‐Demographic Factors

The results showed that medication adherence was significantly associated with sex, distance to health facility, high monthly income, formal education, being employed, age and parity. However, marital status and religion had no association with medication adherence. This is illustrated in Table 5.

**TABLE 5:** Association between medication adherence and socio‐demographic factors.

Socio‐demographic factors	Medication adherence	*p*
	226 (100)	
**Sex**		
Male	140 (61.95)	< 0.001
Female	86 (38.05)	
**Distance to the nearest health facility**		
Less than 2 km	145 (64.16)	< 0.001
More than 2 km	81 (35.84)	
**Religion**		
Islam	84 (37.17)	0.0650
Christian	78 (34.51)	
African Traditional religion	64 (28.32)	
**Monthly income levels**		
Less than GHC 350 (less than minimum wage)	61 (26.99)	< 0.001
More than GHC 351 (more than the minimum wage)	165 (73.01)	
**Educational status**		
No formal education	59 (26.11)	< 0.001
Formal education	167 (73.89)	
**Marital status**		
Married	86 (38.05)	0.0621
Single	58 (25.66)	
Divorced/separated	82 (36.28)	
**Employment status**		
Fully employed	152 (67.26)	< 0.001
Unemployed/retired	74 (32.74)	
**Continuous/interval‐scale variables**		
Age (years)	63.24 ± 16.44	0.002
Parity	5.68 ± 2.89	0.000

*Note:* Data are presented as *p*‐values were determined using chi‐squared for categorical variables and independent *t*‐test for continuous variables.

### 3.6 Association Between Medication Adherence and the Quality of Life of Participants

The association between quality of life and medication adherence was determined, and it was revealed that only the social health dimension of WHOQoL was significantly associated with adherence to medication. The other dimensions of QoL were not associated with medication adherence, as indicated in Table 6.

**TABLE 6:** Association between quality of life (QoL) and medication adherence.

Participants' QoL	Medication adherence	*p*
	226 (100)	
QoL (Total WHOQoL score)	91 (40.27)	0.365
**WHOQoL domains**		
Physical health	68 (30.09)	0.133
Psychological health	90 (39.82)	0.355
Social health	131 (57.96)	0.011
Environmental health	49 (21.68)	0.941

*Note:* Data are presented as *p*‐values were determined using chi‐squared values.

## 4 Discussion

The study revealed that females constituted most of the participants, and this is supported by the findings of a systematic review and meta‐analysis (Bosu and Bosu 2021) in Ghana. This is possibly because in the general population females make up 50.7% of the population and males 49.3%, with a nationwide sex ratio of 97 males for every 100 females (Ghana Statistical Service 2021). It is possible that the prevalence of females in this study is related to the fact that females have been demonstrated to be higher in numbers nationally. In another study on healthcare‐seeking behaviour among rural women in India, the authors (Reddy et al. 2017) established that only one‐third of the individuals sought medical attention as soon as symptoms surfaced, and nearly half of the women needed permission from family members to receive health services. The study accentuated the fact that rural women's healthcare‐seeking behaviour is influenced by socio‐economic circumstances and geographic factors, as their decision regarding which doctor to see was influenced more by the cost of the consultation and the location of the medical facility than by the credentials of the staff and the standard of care delivered (Reddy et al. 2017). This study revealed that over a third of rural PLWHPT had no formal education. It is interesting to note that this is consistent with a previous study that was carried out by Konlan, Afam‐adjei, et al. (2020) and Konlan, Baku, et al. (2020) in an urban setting where over a third of PLWHPT were reported to have had no formal education.

This study found a high rate of medication adherence in more than half of rural PLWHPT. This is akin to studies (Sanuade et al. 2020, 2021; Sitlinger and Zafar 2018) that have equally found a high rate of adherence and, in contrast to other studies (Aziato et al. 2021; Konlan et al. 2023) that found low medication adherence among PLWHPT. This finding is suggestive that the majority of rural people tend to have trust and confidence in the medications being given to them for their high BPs, and this could be responsible for the high level of adherence as compared to their urban counterparts in Ghana, as reported earlier (Aziato et al. 2021; Konlan et al. 2023). The high medication adherence rate observed in this study is positive for hypertension management, as the high medication adherence would result in low complications.

In this study, the majority of rural PLWHPT who did not adhere to the medication reported occasionally forgetting to take their medications with them on trips outside of home or to their workplaces or farms, while some admitted that taking their medications was a real inconvenience, the reason they defaulted. Some also claimed that they did not trust the allopathic drugs and that they preferred herbal medication use. It has been determined that forgetfulness is a major contributor to medication non‐adherence (Al‐Ramahi 2015; Khan et al. 2014). This finding requires that measures are instituted to reduce forgetfulness, such as putting reminder systems in place with the aid of mobile phone technologies. We recommend the vigorous incorporation of mobile health systems in the management of hypertension to overcome the issues of forgetfulness in hypertension management. Anti‐hypertensive medication use is also known to be severely hindered by the inconvenience associated with taking medications out of the home in a study conducted in England (Fraser‐Hurt et al. 2022) and measures that address the challenge of carrying medication, such as providing portable systems for storing and carrying medications, are encouraged particularly in chronic diseased persons.

Age, parity, high monthly income level, formal education and being employed, among others, all had extremely high levels of significance in this study. It is generally known that medication adherence appears to vary depending on a person's age (Fraser‐Hurt et al. 2022). Rural PLWHPT with a high level of drug adherence were noticeably older in this study. Younger individuals' busier lifestyles and greater emphasis on their work and social lives rather than on their illnesses may be a contributing factor to this finding (Fraser‐Hurt et al. 2022). To prevent complications associated with hypertension, treatment compliance is crucial (Fraser‐Hurt et al. 2022) in all age groups, particularly younger persons who will likely live with the disease for longer periods. This study's findings found medication adherence to be highly and positively influenced by age, like a study in Austria (Berner et al. 2019) and contrary to the findings of Ugwuja et al. (2015) who stated older age as a factor in decreased medication adherence rates.

Males were found in this study to be two times more likely to stick to their medication than their female counterparts, indicating that sex was also extremely substantially connected with medication adherence. According to several studies, men are likely to demonstrate greater adherence than women (Zhang et al. 2017; Sanuade et al. 2021). However, findings from other research show that women adhere to anti‐hypertensive medication better than men (Berner et al. 2019; Konlan et al. 2023). The findings in this study could be because of the power dynamics in rural Ghana where the societies are highly male‐dominant, and women might need the consent and authorisation of their male partners even in decisions related to their own health. Further, rural women in Ghana are overwhelmed with domestic activities and could easily forget to take their medications. In addition, because health facilities are far from the communities, most patients require resources to travel to health centres for medications, and these resources are limited among women, and thus could be responsible for the low adherence observed among the females in this study.

Majority of rural people living with hypertension had predominantly a low quality of life. This low quality of life of rural persons living with hypertension means that most of these hypertensives have challenges related to their physical, psychological and environmental health requiring urgent attention to improve their quality of life. This is consistent with a study that found that not a significant proportion of elderly surveyed participants reported having a good quality of life (Reddy et al. 2017). Older people, particularly those with chronic diseases, tend to have deterioration in their quality of life (Aziato et al. 2021). Another study discovered that individuals with hypertension had a lesser quality of life than those without hypertension (Zhang et al. 2017). The low quality of life found in this study calls for pragmatic measures to improve the quality of life of persons living with hypertension in rural Ghana, as hypertension is a life‐long disease and would likely stay with sufferers for a longer time. Exploring measures to improve the quality of life of this unique population from the perspective of the participants would be extremely useful so as to institute measures to improve their quality of life.

The high adherence to BP medication in this study was not significantly associated with any measure of patient overall QoL, in contrast to a study in Vietnam by Ha et al. (2014) that rather showed a significant relationship between treatment adherence and quality of life (QoL) in terms of physical, psychological and environmental health. Another study in Saudi Arabia by Alsaqabi and Rabbani (2020) showed that although poor adherence was linked to decreased overall perceived QoL and health, medication adherence was not significantly correlated with any of the QoL variables. However, medication adherence was found to be only significantly associated with the social health domain of the quality of life. This strong relationship between medication adherence and the social domain of the quality of life is indicative of the role of social connectivity and networks of people in relation to high BP management. Social networks such as family and friends could assist in providing resources for health seeking in rural communities and thus be key in promoting adherence, as found in this study. The findings suggest that participants who adhered to medication tend to have better satisfaction with their personal relationships, a satisfactory sex life and tend to get support from family and friends. This means that people who adhere to treatment were those who enjoyed more support socially and vice versa. Social networks promote treatment adherence as they provide a safe haven to discuss challenges associated with the disease process and serve as channels for voicing‐out concerns regarding treatment (Aziato et al. 2021).

## 5 Conclusion

The results revealed a high rate of treatment adherence, 226 (64%) with low quality of life among the participants. Adherence to treatment was more associated with males as compared to females. The social health dimension of the quality of life was significantly associated with treatment adherence. The role of social networks in enhancing adherence was a major finding in this study and could be harnessed by nurses and midwives to improve the lives of people diagnosed with hypertension. It is recommended that measures that promote remembrance and reduce forgetfulness regarding medication, such as mobile health and digital technologies, be implemented to enhance treatment adherence. Further, measures aimed at improving resource allocation for women in rural communities, such as women's economic empowerment programmes, are encouraged to enhance health‐seeking and treatment adherence. In addition, studies that explore the specific domains of the quality of life of the participants qualitatively are encouraged to identify the unique challenges of this population so that measures aimed at improving their quality of life can be instituted.

## 6 Limitations of the Study

The assessment of the key variables using a questionnaire could have been affected by recall bias even though the investigators entreated the respondents to frankly respond to each question on the questionnaire. Future studies should consider introducing a qualitative aspect to elicit more subjective data. However, the findings in this study provide a basis for in‐depth studies using qualitative designs in future.

The study used a cross‐sectional design, making it difficult to infer causation, but the study provides some evidence about the association between quality of life and treatment adherence, and this can serve as a basis for future longitudinal studies.

This study did not assess the BPs of the participants in order that such data could be compared with the level of adherence, and this is a limitation of the study as the BPs could have provided some objective indication of treatment adherence, as persons who claim to adhere to treatment are expected to have manageable or lower BPs compared to their compatriots. Future studies are encouraged to include this aspect to provide more robust data.

## Author Contributions

A.B.A. contributed to design, data analysis, drafting the manuscript and bears the primary responsibility for the content of the manuscript. J.B.A. was involved in the data collection and proofreading of the manuscript. K.D.K. was involved in the conception of the study and review of the manuscript. G.D. was involved in the design of the study and revised the manuscript. All the authors read and approved the content of the manuscript.

## Ethics Statement

The study data were part of the protocol titled: ‘Treatment adherence, quality of life and coping strategies of rural hypertensives in the Northeast Region of Ghana’ (Protocol ID: CHAGIRB05022022). We obtained permission from the Christian Health Association of Ghana and also we obtained permission from the Management of the chosen Hospital. We also ensured that the data collection was done in conformity with the Helsinki declaration on research ethics. Also, we explained the purpose of the study to each respondent before obtaining written informed consent for the study. Further, we ensured confidentiality of the data collected by assigning unique codes to each response. We also maintained privacy and ensured anonymity throughout the data collection and analysis of the data.

## Consent

The authors have nothing to report.

## Conflicts of Interest

The authors declare no conflicts of interest.

## Data Availability

The data set supporting the conclusion of this study is available for systematic review and meta‐analysis upon request.
